# Challenges of the Application of In Vitro Digestion for Nanomaterials Safety Assessment

**DOI:** 10.3390/foods13111690

**Published:** 2024-05-28

**Authors:** Nádia Vital, Ana Catarina Gramacho, Mafalda Silva, Maria Cardoso, Paula Alvito, Michel Kranendonk, Maria João Silva, Henriqueta Louro

**Affiliations:** 1 National Institute of Health Dr. Ricardo Jorge (INSA), Department of Human Genetics, 1649-016 Lisbon, Portugal; 2 NOVA Medical School, Universidade NOVA de Lisboa, 1169-056 Lisbon, Portugal; michel.kranendonk@nms.unl.pt; 3 Centre for Toxicogenomics and Human Health (ToxOmics), NOVA Medical School, Universidade NOVA de Lisboa, 1169-056 Lisbon, Portugal; 4 National Institute of Health Dr. Ricardo Jorge (INSA), Department of Food and Nutrition, 1649-016 Lisbon, Portugal; 5 REQUIMTE/LAQV, Faculty of Pharmacy, University of Porto, 4050-313 Porto, Portugal; 6 CESAM—Centre for Environmental and Marine Studies, University of Aveiro, 3810-193 Aveiro, Portugal

**Keywords:** INFOGEST, Caco-2, nanomaterials, digestion product, in vitro simulated digestion

## Abstract

Considering the increase in the production and use of nanomaterials (NM) in food/feed and food contact materials, novel strategies for efficient and sustainable hazard characterization, especially in the early stages of NM development, have been proposed. Some of these strategies encompass the utilization of in vitro simulated digestion prior to cytotoxic and genotoxic assessment. This entails exposing NM to fluids that replicate the three successive phases of digestion: oral, gastric, and intestinal. Subsequently, the resulting digestion products are added to models of intestinal cells to conduct toxicological assays, analyzing multiple endpoints. Nonetheless, exposure of intestinal cells to the digested products may induce cytotoxicity effects, thereby posing a challenge to this strategy. The aim of this work was to describe the challenges encountered with the in vitro digestion INFOGEST 2.0 protocol when using the digestion product in toxicological studies of NM, and the adjustments implemented to enable its use in subsequent in vitro biological assays with intestinal cell models. The adaptation of the digestion fluids, in particular the reduction of the final bile concentration, resulted in a reduced toxic impact of digestion products.

## 1. Introduction

Nanomaterials (NM) from various origins, with diverse chemical constituents (e.g., silver, titanium dioxide, silicon dioxide, zinc oxide) have been developed and are increasingly used in food or food contact materials. Despite the multiple benefits that NMs may bring to the food industry, several are currently being re-evaluated for their safety in food applications. For example, the European Food Safety Authority, EFSA, recently classified TiO_2_, a commonly used food additive (E171), as not safe [1]. Silicon dioxide, another common food additive (E551), is currently under re-evaluation by EFSA as a food additive in foods for diverse age groups [2]. The latter takes into consideration the EFSA Guidance on the presence of small particles including nanoparticles [3] and EFSA Guidance on risk assessment of nanomaterials, in which the use of simulated in vitro digestion models is advised [4]. Novel NMs with great potential to be used in food and food contact materials, such as cellulose nanomaterials, have also been investigated before commercialization. Additionally, the widespread use of NMs may also contribute to increased environmental risks, posed by the disposal of food wastes/food packaging materials in landfills, or the ingested NM potentially being excreted and not removed efficiently by wastewater treatments [5]. This may result in the contamination of terrestrial and aquatic ecosystems and the contaminants may eventually reenterg the human food stream via recycled water or through plant or animal uptake.

As indicated, a more comprehensive characterization of NMs’ hazard after human exposure health, particularly through oral ingestion, is currently warranted. As we previously demonstrated for titanium dioxide NM, subtle differences in the physicochemical properties of the same type of NM may lead to different toxicological outcomes [6,7]. The consequences of physiological processes occurring after ingestion, like digestion, may lead to physicochemical modifications of NMs, and may therefore influence their interactions with biomolecules and their biological effects [8,9]. These modifications are crucial to consider when performing in vitro toxicity evaluations of NM in oral exposure scenarios. Regulatory agencies have recognized this issue, recommending the use of in vitro simulated digestion as a method of mimicking human digestion [4]. When used in association with intestinal cell models, in vitro simulated digestion may contribute to innovative methodologies as alternatives to animal experimentation.

The process of in vitro digestion is characterized by a chain of bioreactions that emulate the chemically and physically complex environments of the different compartments (mouth, stomach, intestine) of the gastrointestinal tract (GIT). Ingested NMs move along these different compartments, with different pH conditions and enzymatic environments, before reaching the intestine.

Recently, we reported on the implementation of a harmonized in vitro digestion method, the INFOGEST 2.0 protocol [10,11], which was used either with [12] or without a food matrix [9]. The INFOGEST 2.0 in vitro digestion method was developed by a network of more than 35 countries within the framework of the Cost Action “INFOGEST” and resulted in an international consensus on a set of digestion experimental parameters. These parameters were discussed and justified in detail based on available in vivo physiological data, then used to develop a static in vitro simulation of adult digestion suitable for food [10]. A primary protocol was published by Minekus et al. [11] and subsequently amended and improved (INFOGEST 2.0) to address challenges associated with the original method by including an oral phase and the use of gastric lipase [10]. This method involves the exposure of material under study to three successive digestive phases: oral, gastric, and intestinal [10]. It has been successfully used to assess the bioaccessibility and bioavailability of pharmaceutical compounds and food components [10,11]. However, there is no established experimental procedure for using digested NM products in subsequent in vitro toxicological studies, especially with frequently applied intestinal cell models, such as human colorectal adenocarcinoma (Caco-2) cells or mucus-secreting human colonic cancer (HT29-MTX-E12) cells.

To assess the suitability of incorporating the aforementioned in vitro digestion method into toxicity evaluations using in vitro intestinal cell models, we previously studied titanium dioxide NM and, more recently, cellulose nanomaterials [8,9,13]. With the progressive application of the in vitro digestion method, several experimental challenges were identified. The major challenge was the toxicity of the digestion product per se resulting from the in vitro digestion mixtures in the absence of NM. Several strategies have been recommended to circumvent this inherent toxicity, depending on the purpose of the study, as recently reviewed [14]. Inactivation of digestive enzymes after the in vitro digestion process may be accomplished by raising the reaction pH through the addition of sodium hydroxide (NaOH) or sodium bicarbonate (NaHCO_3_) [15]. However, this may result in undesired NM agglomeration [16]. Enzyme inhibition with different concentrations of commercially available compounds (e.g., Pefabloc^®^ SC) may also be applied [4,14]. Physical separation of the insoluble digestion products formed by enzymes and/or bile salts through centrifugation, dialysis, or ultrafiltration has also been suggested [14]. No cytotoxicity was reported after a 1:3 dilution of the digestion fluids (i.e., without sample), using the enzyme inhibitor Pefabloc^®^ (1 mM) and bile salts (10 mM) in Caco-2 cells when a prior filtration was included in the digestion process [17]. However, such strategies may lower the concentration range by which the NM can be tested, since smaller fractions of NM may be retained and lost during the process. Heat inactivation of enzymes has also been proposed [14]. However, high-temperature conditions may influence the overall NM dynamics and the structure of the NM as well as the release kinetics for bioactive components [18,19].

The objective of the current study is to describe the inherent toxicity encountered when applying the INFOGEST 2.0 in vitro digestion method in toxicological studies. The effect of several factors was verified in modifying the method’s toxicity, such as osmolality, pH, bile salt concentration, and other components of the digestion product. Based on these findings, adjustments to the INFOGEST 2.0 method were implemented, enabling its use in in vitro biological assays with two widely used intestinal cell models.

## 2. Materials and Methods

### 2.1. Chemicals and Reagents

The following reagents were used to prepare the simulated digestion fluids: KCl, CaCl_2_·2H_2_O, NaHCO_3_, NaCl, MgCl_2_·6H_2_O, NaOH (Merck, Darmstadt, Germany), (NH_4_)_2_CO_3_ (Sigma-Aldrich, St. Louis, MO, USA), KH_2_PO_4_, and HCl (J. T. Baker, Center Valley, PA, USA). Pepsin, α-Amylase, bovine bile, pancreatin, and Pefabloc^®^ SC, were purchased from Sigma-Aldrich^®^ (St. Louis, MO, USA). MTT (3-(4,5-dimethylthiazol-2-yl)-2–5-diphenyltetrazolium bromide) and the DCFDA (2′,7′-dichlorofluorescein diacetate) probe were also obtained from Sigma-Aldrich^®^ (St. Louis, MO, USA). All of the reagents for cell culture maintenance were obtained from Thermo Fisher (Waltham, MA, USA).

### 2.2. In Vitro Digestion Protocol

The study used the standardized static INFOGEST 2.0 in vitro digestion protocol [10].

#### 2.2.1. Preparation of Reagents and Test Tubes

Before performing the protocol, the pH parameter was determined in a preliminary experiment by preparing one replicate tube with the relevant amount of sample, enzymes, and bile for the entire digestion process to define the volumes of HCl and NaOH needed for pH adjustments during the digestive phases in the main experiment. Measurements of pH were done with a pH meter (827 pH lab, metrohm) and with an electrode designed for food systems.

The standard units of activity of all the enzymes to be used in the protocol were determined for each new batch of enzymes or after prolonged storage, according to the INFOGEST 2.0 in vitro digestion protocol [10,11].

The bile salt concentration in the bile bovine commercial product was determined with a commercial kit (DiaSys, Merck cat no. 122129990313, Diagnostic System GmbH, Holzheim, Germany) as recommended [10,11]. From the commercially available options for bile salts, bovine bile was selected because its composition is similar to that of human bile [10].

#### 2.2.2. Digestive Fluids Composition

The protocol simulates three phases of digestion—oral, gastric, and intestinal—by adding specific digestion fluids to each phase: simulated salivary fluid (SSF), simulated gastric fluid (SGF), and simulated intestinal fluid (SIF), respectively. Table 1 describes the composition of the simulated digestion fluids used in each phase, with different salts/electrolytes, CaCl_2_·2H_2_O, water, digestive enzymes, and/or bile salt constituents. (For the detailed composition of the salts/electrolytes of each fluid see Supplementary Table S1).

Besides the standard 10 mM of bile salts used in the SIF of the digestion protocol, a new SIF with a 4 mM bile salt concentration was used in the modified digestion protocol (see Section 3).

Initially, either BSA-water (0.05% (wt) of bovine serum albumin in sterile-filtered water) or phosphate buffer saline (PBS) was used in the in vitro digestion protocol. BSA-water is generally used for testing dispersed NM (e.g., TiO_2_ NM), as dictated by the Nanogenotox protocol [20]. PBS was used for the dispersion of cellulose nanomaterials (CNMs) in our laboratory.

Simulated digestion fluids were prepared by sequentially adding to an initial volume of 1 mL of PBS or BSA-water: (i) 1 mL of SSF, mixed in a mechanical shaker for 2 min at 37 °C; (ii) 2 mL of SGF, mixed for 2 h at 37 °C; (iii) 4 mL of SIF, mixed for 2 h at 37 °C. Subsequently, the enzyme inhibitor Pefabloc^®^ SC was added (final concentration 5 mM) to stop enzyme activity [10]. During this in vitro digestion process, the samples underwent a 1:8 dilution. The final solution obtained at the end of the in vitro digestion protocol comprises the sum of all the components added during all steps of the in vitro digestion protocol and is thereafter named the digestion product.

The digestion product was either used immediately (i.e., after a 5-min stabilization at room temperature) or divided into aliquots for snap-freezing in liquid nitrogen and stored at −80 °C. Previous studies showed no difference in toxicity between freshly prepared and frozen samples.

### 2.3. Osmolality and pH

The samples’ pH was measured with pH-indicator strips (Merck, Darmstadt, Germany). The osmolality was measured using an Osmometer K-7400 (Knawer, Berlin, Germany), which was previously calibrated at 0 with water (Nanopure, Warszawa, Poland) and a calibration solution of 300 mOsm/kg H_2_O (milliosmoles per kg of solvent) [9,21].

### 2.4. Intestinal Cell Culture and Exposure

Two human intestinal cell lines, namely Caco-2 cells and HT29-MTX-E12 cells, both obtained from the European Collection of Authenticated Cell Cultures (ECACC, Salisbury, UK), were used. Caco-2 was chosen due to its tissue source (human colorectal adenocarcinoma), characteristics that resemble human enterocytes, and its general use in in vitro toxicity studies. The HT29-MTX-E12 subclone cells were isolated from an HT29 clone obtained from colorectal adenocarcinoma, which was differentiated into mature goblet cells using methotrexate, normally used as a model of the intestinal mucous layer on nanoparticle diffusion, as informed by the cell line description catalog.

Both cell types were cultured individually in complete cell culture media including Dulbecco’s Modified Eagle Medium (DMEM), supplemented with 1% Amphotericin B (0.25 mg/mL), a 1% solution of 10,000 units/mL of penicillin and 10,000 μg/mL of streptomycin, 2.5% HEPES Buffer, and 10–15% fetal bovine serum (FBS). The cells were maintained at 37 °C in an 5% CO_2_ atmosphere.

The digestion product (DIG) of BSA-water or PBS (i.e., without NM) resulting from the INFOGEST procedure was diluted in complete cell culture media (from 0.04 to 53.3% (*v*/*v*)) and used for the subsequent bioassays. As negative controls, BSA-water or PBS, without undergoing the INFOGEST protocol, were used undiluted.

### 2.5. Cell Viability Assay

MTT, one of the most frequently used colorimetric assays to assess cell viability, was performed after 24 h exposure to undigested and digested samples, as previously described [9]. Briefly, Caco-2 or HT29-MTX-E12 cells were cultured in 96-well plates (2 × 10^4^ cells per well) and incubated for 24 h at 37 °C and 5% CO_2_. The cells were then exposed for 24 h to the digestion product. After exposure, the cells were washed with PBS and incubated with MTT (0.5 mg/mL) for 3 h. The MTT solution was removed and DMSO was added to each well and incubated for 30 min, under constant stirring, at room temperature and protected from light. Absorbance was measured in a Multiskan Ascent Spectrophotometer (Thermo LabSystems, Waltham, MA, USA) at 570 nm (reference filter: 690 nm). The relative viability (%) of the treated cells was expressed as the percentage of absorbance compared to the control (untreated) cells (100% viability). Three independent experiments were performed per exposure condition. Sodium dodecyl sulfate (SDS, 0.01%, for 1 h) was used as a positive control.

### 2.6. Intracellular Reactive Oxygen Species (ROS)

Intracellular reactive oxygen species (ROS) levels were determined with the DCFDA probe, after 3 and 24 h exposure to the digestion product, as previously described [8]. Briefly, Caco-2 and HT29-MTX-E12 cells were seeded in 96-well plates (2 × 10^4^ cells per well, 100 μL/well), using complete cell culture medium, and incubated for 24 h at 37 °C and 5% CO_2_. The cells were then incubated for 30 min with 20 μM of DCFDA, in the dark, at 37 °C. Then the probe solution was removed, and fresh medium containing the different concentrations of digestion product was added, in three replicates. The 2,7-dichlorofluorescein (DCF) fluorescences were determined at excitation 485 nm and emission 535 nm wavelengths, using a SpectraMax ID3 (Molecular Devices, San Jose, CA, USA). The data are reported as relative fluorescence units (RFU) and relative ROS levels expressed as a mean fluorescence ratio (fluorescence of exposed cells/fluorescence of unexposed control from the same experiment). Hydrogen peroxide solution (250 µM, 1 h incubation) was used as a positive control for ROS induction.

### 2.7. Statistical Analysis

The statistical analysis was performed using Prism software (5, GraphPad, San Diego, CA, USA). Provided that the data followed a normal distribution, statistical comparisons of the MTT assay data between treated and control cells were performed, applying a one-way analysis of variance (ANOVA). When necessary, the ANOVA was followed by Dunnett´s post hoc tests to analyze for differences between the different concentrations and the negative control. A two-tailed Student’s *t*-test was used for ROS analysis, for the comparison of digested samples with their respective controls. Non-parametric tests such as the Kruskal–Wallis or the Mann–Whitney U test were applied in all other cases. Differences with a *p*-value lower than 0.05 were considered statistically significant.

## 3. Results and Discussion

### 3.1. Cytotoxicity of the Digestion Product on Intestinal Cells

**Figure 1:**
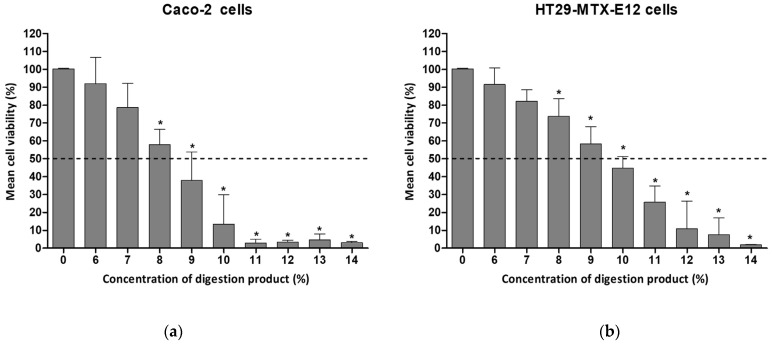
Cell viability of (**a**) Caco-2 cells or (**b**) HT29-MTX-E12 cells, after 24 h exposure to different concentrations of digestion product (0–14%) using the MTT assay. Results are presented as relative viability (mean ± standard deviation, N = 3). *—*p* < 0.0001, One-Way ANOVA, Post Hoc Dunnett’s Multiple Comparison Test. Dashed line indicates 50% viability.

Twenty-four hours of exposure of the intestinal cells to concentrations of digestion product above 8% resulted in significantly reduced levels of viability in both cell types, with Caco-2 presenting 58.9% viability in complete culture medium, as depicted in Figure 1. In HT29-MTX- E12, exposure to 9% digestion product led to 58% cell viability.

A concentration of 7% showed approximately 80% viability for both cell lines (78.6% and 82.2% for Caco-2 and HT29-MTX-E12, respectively). A 1:10 dilution with complete cell culture medium (i.e., 10%) was cytotoxic.

Comparable cytotoxic effects related to the digestion product have also been reported by other authors using Caco-2 cells and the SIF fraction only. A 1:10 dilution of SIF with DMEM medium decreased Caco-2 cells’ viability to 65% after 24 h of exposure [22]. The same SIF dilution, using MEM supplemented with 20% FBS, led to 30.5% viability of Caco-2 cells when exposed for 24 h [23]. The same authors, using a Caco-2/HT29-MTX co-culture model, reported a viability of 86.5% after 24 h exposure to 1:10 dilutions of simulated digestive fluids in the absence of the test compound, and 10.7% viability for a 1:6 dilution. When the exposure time was reduced (4 h), cell viability was much less affected (97.1%) [23].

The need to dilute digestion products to at least a 1:10 dilution factor to maintain low toxicity levels, as indicated by the present work and by others [22,23], narrows the concentration range of NM to be tested in subsequent assays, limited by the initial concentrations of the NM before the digestion procedure. It is worth noting that initial sample concentrations already undergo a 1:8 dilution as part of the application of the INFOGEST protocol. In our previous study, we worked on preventing toxicity induced by the digestion product using sample dilutions, limiting the tested NM concentrations to a maximum of 14.3 µg/mL [8].

The roles of several components of the digestion procedure were verified and are described in the following sections. Results are expected to aid the adaptation of the in vitro digestion procedure to circumvent the high toxicity levels demonstrated by the digestion components. This will allow the use of a more extended range of concentrations of in vitro digested NM samples for toxicity analysis using different endpoints.

### 3.2. Osmolality and pH

Although using a different digestion method, Deloid et al. reported the toxicity of the digestion product and attributed this to the high osmolarity and bile concentrations [24]. Therefore, the roles of these two parameters in the toxicity of the INFOGEST approach were tested. The digestion product dilutions showed pH values of approximately 7 (see Supplementary Figure S1) (compatible with mammalian cell culture [25]), regardless of the percentage of the digestion product. This result indicates the sufficient buffer capacity of the used culture medium to maintain the physiological pH.

The adequate osmolality of cell culture media for use with vertebrate cell lines is between 260 to 320 mOsm/kg, set to mimic the one from serum: 290 mOsm/kg [25]. The encountered osmolality of the different dilutions of digestion product was in the range of 301–309 mOsmol/Kg (see Table 2). Thus, the osmolality was in agreement with the reference values for human cells, although the appropriate osmotic conditions for Caco-2 cells have been indicated to be 336 mOsm/L [26]. As such, the high toxicity observed with the digestion product is highly unlikely to be due to altered pH or osmolality.

### 3.3. The Role of Pefabloc^®^ SC in the Toxicity of the Digestion Product

Pefabloc^®^ has been suggested as a potential cause of the toxicity of the digestion product in different cellular lines, including Caco-2 cells, as recently revised [14]. This is a broad-spectrum trypsin-like serine proteases inhibitor usually applied at the end of the intestinal phase to prevent over-digestion by trypsin and chymotrypsin [10,14]. In its absence, the activity of these enzymes in the digestion product may cause cytotoxic effects by directly degrading human cells.

**Figure 2:**
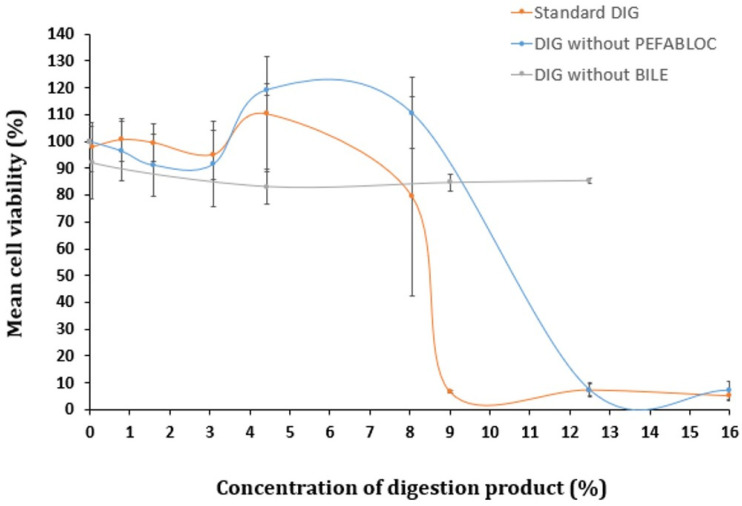
Cell viability of Caco-2 cells undergoing a 24 h exposure to a concentration gradient (%) of digestion product, using the standard in vitro digestion protocol (DIG) or lacking (i) Pefabloc^®^ SC or (ii) bovine bile in its composition, determined by the MTT assay. Results are presented as relative viability (mean ± standard deviation; N > 3).

To determine if Pefabloc^®^ SC plays a role in the digestion product’s toxicity, cells were exposed to the digestion product in the presence or absence of this inhibitor (for reference, in Figure 2, 16% of digestion product contains 0.8 mM of Pefabloc^®^ SC). The comparison of the cytotoxicity of the digestion product with and without Pefabloc^®^ SC did not show significant differences (see Figure 2). This suggests that Pefabloc^®^ does not seem to play a significant role in the toxicity observed with the digestion product using standard settings.

Xavier et al. described no decreased viability of cells of Caco-2/HT29-MTX co-cultures, after a 24 h exposure to 5 mM Pefabloc^®^ SC (with up to a 1:6 dilution in culture medium, corresponding to a final concentration of 0.8 mM Pefabloc^®^ SC), in the absence of simulated digestion fluids [23]. In contrast, when Caco-2/HT29-MTX co-cultures were exposed to the same concentration of Pefabloc^®^ SC together with simulated digestion fluids, their viability was considerably reduced; it fell to 10.7% [23]. The additional use of the PLUS additive (a Pefabloc protector solution) was indicated to avoid covalent attachment to proteins when used in tests with prolonged incubation times at alkaline pH, avoiding cell toxicity [23]. Moreover, recent studies suggest that reducing the Pefabloc^®^ final concentration to 1 mM while maintaining the final bile salt concentration at 10 mM in the digestion product, further diluted to either 1:3 (33.3%) or 1:100 (1%), resulted in no toxicity in Caco-2 cells after a 2 h exposure [27,28]. A reduction to 0.5 mM Pefabloc SC^®^ was recently suggested, based on revised literature, including in vitro epithelial absorption studies [14].

### 3.4. Bile Salts’ Effect on Digestion Product Cytotoxicity

Several authors proposed the concentration of bile salts as the culprit of the cytotoxicity observed with the digestion product [23,24,29]. To investigate this hypothesis, a digestion-adapted procedure was followed, excluding bovine bile from SIF. Toxicity levels were compared with those observed when using the standard digestion protocol. Omitting bile salts from the digestion fluids did not result in significant cytotoxic effects up to 12.5% of digestion product diluted in cell culture medium (Figure 2). However, when bile was included (standard DIG), pronounced cytotoxicity was observed above 9% of the digestion product. Therefore, the presence of bile salts appears to be a major contributor to the toxicity of the digestion products.

Despite this observation, our previous study on titanium dioxide NM did not include any modifications to the standard protocol, since a low concentration range of the digested NM (0.14–14.3 μg/mL), was considered more physiologically relevant, keeping the content of the digestion product below 8% [8,9]. However, in other investigations, in which higher levels of exposures to NM are considered to be more relevant, low concentrations of digestion fluids (<8%) are not feasible. Therefore, further optimization was pursued to explore the possibility of using more moderate concentrations of bovine bile.

During human digestion, bile salts are required for the emulsion, digestion, and absorption of lipids and lipophilic components [30], their release being differentially induced by the diet and presence of specific food components. Bile salts are considered to mediate the disruption of cell membranes via their surfactant-like activity, particularly when the concentration exceeds the critical micellar concentration range [29,31]. In adults, concentrations of bile salts in the lumen of the small intestine range from approximately 3 mM to 20 mM, i.e., in a fast to a fed state, respectively [32,33]. A large variability and an asymmetric distribution of bile salt concentrations are usually observed in the fast state (0.03 to 36.18 mM), but most of these salts are present in concentrations below 5 mM [33]. In the fed state, bile salt concentrations tend to decrease with time subsequent to meal intake [32]. Considering that this current study was performed in the absence of a food matrix (i.e., simulating a fast state), the bile salt concentration was reduced to the still physiologically relevant concentration of 4 mM, in the attempt to minimize the cytotoxicity of the digestion fluids. A similar strategy has been followed by other authors [24,34].

**Figure 3:**
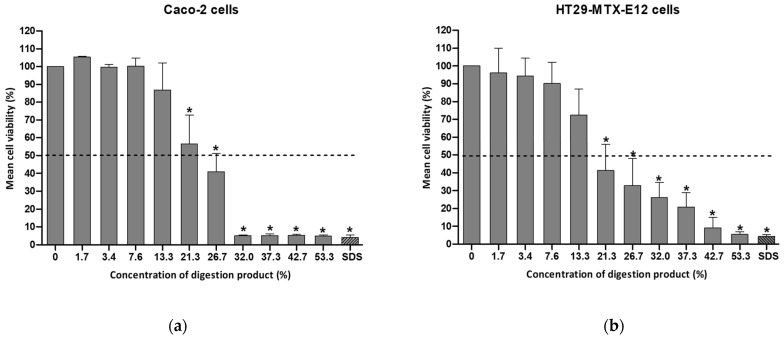
Cell viability of (**a**) Caco-2 cells or (**b**) HT29-MTX-E12 cells, after a 24 h exposure to a concentration range (0–53.3%) of the modified digestion product, containing 4 mM bile salts, determined by the MTT assay. Results are presented as relative viability (mean ± standard deviation; N = 3). *: *p* < 0.0001, One-Way ANOVA, Post Hoc Dunnett’s Multiple Comparison Test). Positive control SDS (sodium dodecyl sulfate: 0.01%, for 1 h). Dashed line indicates 50% viability.

The modified digestion product containing 4 mM bile salts was tested for cytotoxicity using either Caco-2 or HT29-MTX-E12 cells (see Figure 3).

By reducing the bile salt concentration to 4 mM, the viability of Caco-2 cells after a 24 h exposure to a 13% concentration of digestion product was above 70% (86.9% viability), as illustrated in Figure 3. In the case of HT29-MTX-cells the viability was 72.3%. This adjustment enables an increase in the percentage of the modified digestion product used in cell culture medium (7–8% in the standard procedure versus 13% in the modified protocol). This consequently enables the extension of the maximum NM concentration to be tested. Cell viability dropped significantly at concentrations of the digestion product exceeding 20% (see Figure 3).

The study by Araiza-Calahorra et al. reported approximately 60% and 55% viability of Caco-2 cells even after a 2 h incubation with the digestion product constituted by SGF and SIF containing bile salts at final concentrations between 1 and 2 mM, respectively [29]. These authors used an altered INFOGEST digestion protocol, omitting the oral step (SSF), increasing the SIF incubation time from 2 h to 3 h at pH 6.8, and quenching the pepsin activity with 0.2 M sodium bicarbonate to reach a final pH of 7.0. Two additional experiments were performed with a digestion product containing lower concentrations of bile salts in Caco-2 cells: 1 mM and 2 mM (unpublished results ). After exposure to 32% of each of the digestion products, less than 70% Caco-2 viability was observed. From this concentration onward, the viability of the digestion product with 1 mM was maintained at around 70%, while less than 10% viability was observed after testing with a 53.3% concentration of the digestion product (the maximum concentration tested) containing 2 mM. However, using a bile concentration below 4 mM might not represent appropriately the physiological digestion state, thus it was decided to use this concentration.

### 3.5. Modified Digestion Product Induces Reactive Oxygen Species

**Figure 4:**
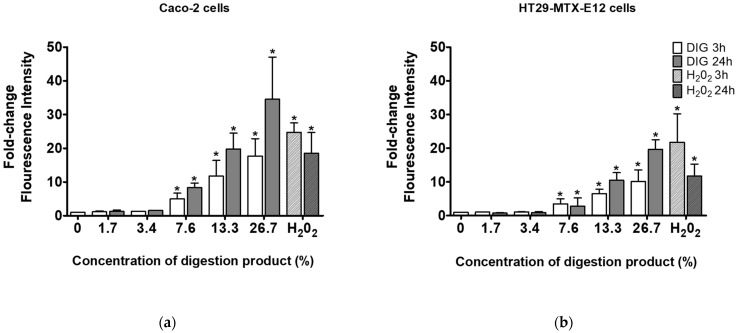
ROS generation in (**a**) Caco-2 cells or (**b**) HT29-MTX-E12 cells, after 3 and 24 h exposure to different concentrations (0–26.7%) of modified digestion product, using 4 mM bile (0: negative control, medium only). *—Significantly different from the negative control (*p* < 0.05, Student’s *t*-test).

A study by Barrasa et al. (2013) reported that bile salts exert cytotoxic effects by increasing the production of reactive oxygen/nitrogen species (ROS/RNS) and the subsequent apoptosis triggered by oxidative stress and membrane damage [35]. Therefore, the ROS generation was investigated after the cells were exposed to the modified digestion product with 4 mM of bile salts (Figure 4). When exposed to digestion fluid concentrations of 7.6% and above, a significant concentration-dependent increase in ROS formation was observed, indicative of oxidative stress under the modified digestion conditions. Interestingly, no reduction of cell viability was observed after exposures to 7.6% and 13.3% of digestion fluids (see Figure 3). This indicates some cellular tolerance to ROS and warrants caution in the interpretation of toxicological findings after using the standardized in vitro simulated digestion. This initial tolerance suggests the need to include appropriate negative controls in the digestion protocol, using solely the digestion product at concentrations equivalent to those used when testing in vitro digested NM samples for toxicological assays.

## 4. Conclusions

Given the increase in production and use of NM in multiple applications such as in the food/feed and food contact materials industries, a more elaborate hazard evaluation is needed. This, in combination with testing policies seeking to reduce animal use, requires strategies including in vitro digestion coupled with informative in vitro cyto- and genotoxic assessment. This could be of particular interest for application during the early stages of NM development, allowing the introduction of hazard-mitigating procedures during NM design. However, the cytotoxicity of digestive fluids used in the in vitro digestion simulation methods on cultured intestinal epithelial cells remains a major hurdle in the way of the use of in vitro digestion in subsequent cyto- and genotoxicity testing. Thus, currently, a standardized protocol to be used for toxicological studies is still needed. To support that achievement, future studies are needed to better understand the toxic effects of the digestion product components on different intestinal models. For example, testing different concentration ranges of bile from different origins (e.g., porcine or bovine) combined with different concentration ranges of Pefabloc or other enzyme inhibitors, taking into consideration possible interactions between those components, either with or without a food matrix.

Considering the significance of the standardized INFOGEST in vitro digestion method and its role in enhancing regulatory decisions and facilitating a more accurate safety assessment of NM, findings from the present study prompt us to make several recommendations. These recommendations are:

1. In toxicology testing, include a digestion product control, corresponding to each concentration assayed when evaluating digested NM;
2. Conduct preliminary experiments to assess the sensitivity of the selected cell model and culture system to the digestion product;
3. When feasible, start the digestion process with the highest attainable concentration of the test material, enabling maximum dilution levels of both the sample and the digestion product before testing;
4. Consider reducing the bile salts concentration in the digestion product, particularly when the experiment aims to replicate a fasting-diet state.

These recommendations are currently being explored in ongoing research using the present study conditions, i.e., bile at 4 mM in the digestion fluids, and they enable testing of a reasonable concentration range of the NM under study in our laboratory while mimicking physiological fasting conditions.

In addition to these recommendations, in the context of safety assessments of nanomaterials, the specific type and structure of the nanomaterials under analysis should additionally be carefully considered.

These recommendations are expected to assist other researchers in designing in vitro approaches that include a digestion step, allowing them to mitigate the toxicity of the digestion product while preserving the method´s physiological relevance.

## Figures and Tables

**Table 1:** Composition of simulated digestion fluids added at each phase of the standardized INFOGEST 2.0 in vitro digestion method.

Oral Phase (pH7) Simulated Salivary Fluid (SSF, 1×)	Gastric Phase (pH3) Simulated Gastric Fluid (SGF, 1×)	Intestinal Phase (pH7) Simulated Intestinal Fluid (SIF, 1×)
– 1.25× electrolyte stock solution;	– 1.25× electrolyte stock solution;	– 1.25× electrolyte stock solution;
– CaCl_2_·2H_2_O: 1.5 mM;	– CaCl_2_·2H_2_O: 0.15 mM;	– CaCl_2_·2H_2_O: 0.6 mM;
– α-amylase: 75 U/mL;	– Pepsin: 2000 U/mL;	– bovine bile: 10 mM;
– Milli-Q water.	– Milli-Q water.	– Pancreatin: 100 U/mL (in trypsin activity);
		– Milli-Q water.

**Table 2:** Osmolality results of DIG BSA-water diluted in complete cell culture medium.

Digestion Product (%)	Osmolality (mOsmol/kg)
1.6	305
3.1	306
6.3	309
12	301

## Data Availability

The original contributions presented in the study are included in the article/supplementary material, further inquiries can be directed to the corresponding author.

## Supplementary Materials

The following supporting information can be downloaded at: https://www.mdpi.com/article/10.3390/foods13111690/s1, Table S1. Composition of electrolyte stock solution of each simulated digestion fluids used in the in vitro digestion method (concentration 1.25X). For additional details, see elsewhere [10]. Figure S1. Results of pH determination of different concentrations of the unmodified digestion product (with 10 mM bile salts) diluted in cell culture medium (0–15%). The 100% mark corresponds to the digestion product directly used for exposure of the cells, without dilution in the culture media.

## References

[1] Younes M., Aquilina G., Castle L., Engel K.H., Fowler P., Frutos Fernandez M.J., Fürst P., Gundert-Remy U., Gürtler R., EFSA Panel on Food Additives and Flavourings (FAF) (2021). Scientific opinion on the safety assessment of titanium dioxide (E171) as a food additive. EFSA J..

[2] Castle L., Degen G., Engel K.H., Frutos Fernández M.J., Remy U.G., Gürtler R., Husoy T., Manco M., Mennes W., EFSA Scientific Panel on Food Additives and Flavourings (FAF) In Proceedings of the 41st FAF Panel Meeting, 14–15 November 2023. https://www.efsa.europa.eu/sites/default/files/2023-12/141123-m.pdf.

[3] More S., Bampidis V., Benford D., Bragard C., Halldorsson T., Hernández-Jerez A., Bennekou S.H., Koutsoumanis K., Lambré C., EFSA Scientific Committee (2021). Guidance on technical requirements for regulated food and feed product applications to establish the presence of small particles including nanoparticles. EFSA J..

[4] More S., Bampidis V., Benford D., Bragard C., Halldorsson T., Hernández-Jerez A., Bennekou S.H., Koutsoumanis K., Lambré C., EFSA Scientific Committee (2021). Guidance on risk assessment of nanomaterials to be applied in the food and feed chain: Human and animal health. EFSA J..

[5] Bolan S., Sharma S., Mukherjee S., Zhou P., Mandal J., Srivastava P., Hou D., Edussuriya R., Vithanage M., Truong V.K. (2024). The distribution, fate, and environmental impacts of food additive nanomaterials in soil and aquatic ecosystems. Sci. Total Environ..

[6] Louro H., Saquib Q., Faisal M., Al-Khedhairy A., Alatar A. (2018). Relevance of physicochemical characterization of nanomaterials for understanding nano-cellular interactions. Cellular and Molecular Toxicology of Nanoparticles.

[7] Louro H., Saruga A., Santos J., Pinhão M., Silva M.J. (2019). Biological impact of metal nanomaterials in relation to their physicochemical characteristics. Toxicol. Vitr..

[8] Vieira A., Vital N., Rolo D., Roque R., Gonçalves L.M., Bettencourt A., Silva M.J., Louro H. (2022). Investigation of the genotoxicity of digested titanium dioxide nanomaterials in human intestinal cells. Food. Chem. Toxicol..

[9] Bettencourt A., Gonçalves L.M., Gramacho A.C., Vieira A., Rolo D., Martins C., Assunção R., Alvito P., Silva M.J., Louro H. (2020). Analysis of the characteristics and cytotoxicity of titanium dioxide nanomaterials following simulated in vitro digestion. Nanomaterials.

[10] Brodkorb A., Egger L., Alminger M., Alvito P., Assunção R., Ballance S., Bohn T., Bourlieu-Lacanal C., Boutrou R., Carrière F. (2019). INFOGEST static in vitro simulation of gastrointestinal food digestion. Nat. Protoc..

[11] Minekus M., Alminger M., Alvito P., Balance S., Bohn T., Bourlieu C., Carrière F., Boutrou R., Corredig M., Dupont D. (2014). A standardised static in vitro digestion method suitable for food—An international consensus. Food. Funct..

[12] Assunção R., Martins C., Dupont D., Alvito P. (2016). Patulin and ochratoxin A co-occurrence and their bioaccessibility in processed cereal-based foods: A contribution for Portuguese children risk assessment. Food Chem. Toxicol..

[13] Vital N., Ventura C., Kranendonk M., Silva M.J., Louro H. (2022). Toxicological assessment of cellulose nanomaterials: Oral exposure. Nanomaterials.

[14] Kondrashina A., Arranz E., Cilla A., Faria M.A., Santos-Hernández M., Miralles B., Hashemi N., Rasmussen M.K., Young J.F., Barberá R. (2023). Coupling in vitro food digestion with in vitro epithelial absorption; recommendations for biocompatibility. Crit. Rev. Food Sci. Nutr..

[15] Mulet-Cabero A.I., Egger L., Portmann R., Ménard O., Marze S., Minekus M., Le Feunteun S., Sarkar A., Grundy M.M., Carrière F. (2020). A standardised semi-dynamic in vitro digestion method suitable for food—An international consensus. Food Funct..

[16] Madalena D., Fernandes J.M., Avelar Z., Gonçalves R.F.S., Ramos Ó.L., Vicente A.A., Pinheiro A.C. (2022). Emerging challenges in assessing bio-based nanosystems’ behaviour under in vitro digestion focused on food applications—A critical view and future perspectives. Food Res. Int..

[17] Gonçalves R.F.S., Martins J.T., Abrunhosa L., Baixinho J., Matias A.A., Vicente A.A., Pinheiro A.C. (2021). Lipid-based nanostructures as a strategy to enhance curcumin bioaccessibility: Behavior under digestion and cytotoxicity assessment. Food Res. Int..

[18] Pinheiro A.C., Gonçalves R.F.S., Madalena D.A., Vicente A.A. (2017). Towards the understanding of the behavior of bio-based nanostructures during in vitro digestion. Curr. Opin. Food Sci..

[19] Li C., Yu W., Wu P., Chen X.D. (2020). Current in vitro digestion systems for understanding food digestion in human upper gastrointestinal tract. Trends Food Sci. Technol..

[20] Jensen A.K., Kembouche Y., Christiansen E., Jacobsen N.R., Wallin H., Guiot C., Spalla O., Witschger O. (2011). Towards a Method for Detecting the Potential Genotoxicity of Nanomaterials: Final Protocol for Producing Suitable Manufactured Nanomaterials Exposure Media Report; The Generic NANOGENOTOX Dispersion Protocol Standard Operation Procedure (SOP) and Background Documentation.

[21] Gramacho A. (2019). Assessment of the Cellular Effects of Ingested Titanium Dioxide Nanomaterials in Intestinal. Master’s Thesis.

[22] Gil-Sánchez I., Monge M., Miralles B., Armentia G., Cueva C., Crespo J., López-de-Luzuriaga J.M., Olmos M.E., Bartolomé B., Llano D.G. (2018). Some new findings on the potential use of biocompatible silver nanoparticles in winemaking. Innov. Food Sci. Emerg. Technol..

[23] Xavier M., Rodrigues P.M., Neto M.D., Guedes M.I., Calero V., Pastrana L., Gonçalves C. (2023). From mouth to gut: Microfluidic in vitro simulation of human gastro-intestinal digestion and intestinal permeability. Analyst.

[24] DeLoid G.M., Wang Y., Kapronezai K., Lorente L.R., Zhang R., Pyrgiotakis G., Konduru N.V., Ericsson M., White J.C., De La Torre-Roche R. (2017). An integrated methodology for assessing the impact of food matrix and gastrointestinal effects on the biokinetics and cellular toxicity of ingested engineered nanomaterials. Part. Fibre Toxicol..

[25] Freshney R.I. (2010). Culture of Animal Cells: A Manual of Basic Technique and Specialized Applications.

[26] Grauso M., Lan A., Andriamihaja M., Bouillaud F., Blachier F. (2019). Hyperosmolar environment and intestinal epithelial cells: Impact on mitochondrial oxygen consumption, proliferation, and barrier function in vitro. Sci. Rep..

[27] Arranz E., Segat A., Velayos G., Flynn C., Brodkorb A., Giblin L. (2023). Dairy and plant-based protein beverages: In vitro digestion behaviour and effect on intestinal barrier biomarkers. Food Res. Int..

[28] Gonçalves R.F.S., Martins J.T., Abrunhosa L., Vicente A.A., Pinheiro A.C. (2021). Nanoemulsions for enhancement of curcumin bioavailability and their safety evaluation: Effect of Emulsifier Type. Nanomaterials.

[29] Araiza-Calahorra A., Wang Y., Boesch C., Zhao Y., Sarkar A. (2020). Pickering emulsions stabilized by colloidal gel particles complexed or conjugated with biopolymers to enhance bioaccessibility and cellular uptake of curcumin. Curr. Res. Food Sci..

[30] Monte M.J., Marin J.J., Antelo A., Vazquez-Tato J. (2009). Bile acids: Chemistry, physiology, and pathophysiology. World J. Gastroenterol..

[31] Marin J.J., Macias R.I., Briz O., Banales J.M., Monte M.J. (2015). Bile acids in physiology, pathology and pharmacology. Curr. Drug Metab..

[32] Kalantzi L., Goumas K., Kalioras V., Abrahamsson B., Dressman J.B., Reppas C. (2006). Characterization of the human upper gastrointestinal contents under conditions simulating bioavailability/bioequivalence studies. Pharm. Res..

[33] Riethorst D., Mols R., Duchateau G., Tack J., Brouwers J., Augustijns P. (2016). Characterization of human duodenal fluids in fasted and fed state conditions. J. Pharm. Sci..

[34] Santos-Hernández M., Amigo L., Recio I. (2020). Induction of CCK and GLP-1 release in enteroendocrine cells by egg white peptides generated during gastrointestinal digestion. Food Chem..

[35] Barrasa J.I., Olmo N., Lizarbe M.A., Turnay J. (2013). Bile acids in the colon, from healthy to cytotoxic molecules. Toxicol. Vitr..

