# Supplementary material for: Challenges of the Application of In Vitro Digestion for Nanomaterials Safety Assessment
Source: Foods. 2024 May 28;13(11):1690. doi: 10.3390/foods13111690 (PMC11171843; doi:10.3390/foods13111690)
Supplement: Supplementary file 1 [file foods-13-01690-s001.zip › foods-3003093-supplementary.pdf]

foods

MDPI

Article

Challenges of the Application of in vitro Digestion for Nanomaterials Safety Assessment

Nádia Vital [1,2,3], Ana C. Gramacho [1], Mafalda Silva [4,5], Maria Cardoso [1], Paula Alvito [4,6], Michel Kranendonk [2,3], Maria João Silva [1,3], and Henriqueta Louro [1,3*]

Supplementary Materials:

Table S1. Composition of electrolyte stock solution of each simulated digestion fluids used in the in vitro digestion method (concentration 1.25X). For additional details, see elsewhere [10].

Salt solution	SSF Final salt concentration (mmol/L)	SGF Final salt concentration (mmol/L)	SIF Final salt concentration (mmol/L)
$CaCl_2{\cdot}2H_2O$*	1.5	0.15	0.6
HCl	1.1	15.6	8.4
KCl	15.1	6.9	6.8
KH_2PO_4	3.7	0.9	0.8
$MgCl_2{\cdot}6H_2O$	0.15	0.12	0.33
NaCl	-	47.2	38.4
$NaHCO_3$	13.6	25	85
$(NH_4)_2CO_3$	0.06	0.5	-

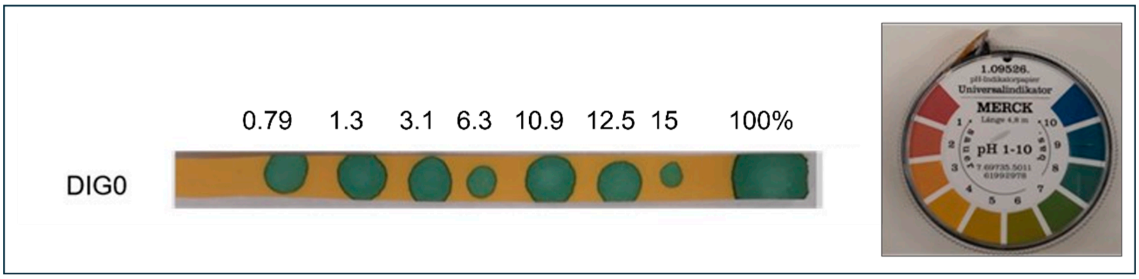

Figure S1. Results of pH determination of different concentrations of the unmodified digestion product (with 10 mM bile salts) diluted in cell culture medium (0-15 %). The 100% mark corresponds to the digestion product directly used for exposure of the cells, without dilution in the culture media.
